# Patterns of salinity regime in coastal lakes based on structure of benthic invertebrates

**DOI:** 10.1371/journal.pone.0207825

**Published:** 2018-11-26

**Authors:** Krystian Obolewski, Katarzyna Glińska-Lewczuk, Monika Szymańska, Natalia Mrozińska, Martyna Bąkowska, Aleksander Astel, Sylwia Lew, Ewa Paturej

**Affiliations:** 1 Departament of Hydrobiology, Kazimierz Wielki University, Bydgoszcz, Poland; 2 Department of Water Resources, Climatology and Environmental Management, University of Warmia and Mazury, Olsztyn-Kortowo, Poland; 3 Department of Environmental Chemistry, Pomeranian University in Słupsk, Słupsk, Poland; 4 Department of Microbiology, University of Warmia and Mazury, Olsztyn-Kortowo, Poland; 5 Department of Tourism, Recreation and Ecology, University of Warmia and Mazury, Olsztyn-Kortowo, Poland; Universitat de Barcelona, SPAIN

## Abstract

The macrozoobenthic diversity patterns along a brackish–freshwater salinity gradient have been identified, considering effects of differences in the level of hydrological connection of coastal lakes with the sea on the structure of benthic invertebrate communities. The study is based on samples from six coastal lakes located along the southern coast of the Baltic Sea in Poland. The analysis of environmental and biological data confirmed the existence of stable phases (brackish water vs. freshwater), but as a result of periodical intrusion of seawater, adaptation of animal communities takes place, which was reflected in low values of the predictors describing them (number of taxa, density and diversity). Redundancy analysis indicates that values of conductivity and salinity are the major factors that determine the abundance of dominant groups of benthic fauna. The gradient of hydrological connection of the lakes with the sea accounted for 50% of the variance in biological data, physico-chemical variables for 25%, trophic variables for 15%, and only 9% of the variance was unexplained. The major implication of our results is that coastal lakes that differ only slightly in salinity can have alternative, regional patterns of diversity of structure of benthic fauna. Periodical inflow of brackish waters initiates adaptive cycles of benthic fauna, and their frequency is strongly linked with the hydrological regime. The rhythm of the inflow of seawater is variable, so that management and protection of coastal lakes are extremely complicated.

## Introduction

Recent studies on the structure of benthic invertebrate communities have reported regime shifts in ecosystems [1, 2]. Initially, the shifts were studied in shallow northern freshwater lakes, which at moderately high phosphorus concentrations can maintain two alternative phases: turbid or clear [3–6]. The clear-water phase is characterized by a high abundance of submerged vegetation, a high proportion of predatory fish, and a high zooplankton: phytoplankton (Z:P) biomass ratio, while in the turbid phase, submerged vegetation is absent, or its abundance is low, very much like the proportion of predatory fish and Z:P ratio [7]. Currently this approach is extended to other lake types, including coastal water bodies varying in the degree of hydrological connection with the sea and, consequently, in salinity [8].

Coastal freshwater and brackish water bodies are important ecotones worldwide, more and more often threatened by human impact, including the rising sea level and activity of the increasing human population [9, 10]. Simultaneously, because of their geographic location between the sea and land, lagoons and coastal lakes are the most productive and complicated marine ecosystems [11]. Their functioning is closely related to hydrological conditions and the degree of intrusion of seawater [8, 12, 13]. Moreover, it is believed that permanent intrusion or a complete lack of intrusion play a crucial role in the hydrological regime of shallow lagoons and coastal lakes, by ensuring the stability and functioning in feedback mechanisms for aquatic organisms [8, 14]. There is relatively little information on ecosystems with periodical intrusion, as dynamic systems, where reaching a permanent balance is impossible according to the theory of ecosystem resilience [15].

More and more studies show that biological components are important for determination of the true environmental state [16, 17] and assessment of the integrity of biological systems associated with the sea [18, 19]. This results from the evolution of those ecosystems from open embayment to barrier-lagoons progressively filled with seawater, and lakes completely isolated from the sea [12]. In water bodies located close to an oceanic estuaries analyses can be performed at each of the phases [8], but in the case of small, closed seas, e.g. the Baltic Sea, need analyse stabilized systems of hydrological connection of lakes with the sea [20]. Hence, in this group of water bodies, three major types can be distinguished: permanently connected with the sea by canals or river mouths, with periodical influx of brackish water (unblocking of blocked canals), and permanently isolated. Only their complete isolation or permanent openness lead to stability of environmental conditions (e.g. salinity, temperature, oxygen content, trophic state) in contrast to water bodies with periodical intrusion of seawater [11]. In the latter case, environmental conditions are characterized by remarkable variation in time and space, fluctuation of biological productivity, and periodical exchange with the neighbouring marine ecosystem [19, 21]. According to the [22], benthic invertebrates are among the suggested biological elements used for evaluation of water status [23], because this group is generally considered as a potentially sensitive indicator of ecosystem health [24]. The observed changes in structure of benthic fauna may also indicate environmental stresses, which makes them excellent integrators of changes in aquatic ecosystems hydrologically linked with one another [25, 26]. Besides, both physical and biological processes can proceed differently in transitional zones, so groups of benthic fauna react differently, too the benthic macrofauna plays a major role in the food cycle, decomposition of detritus, and as a source of food for higher trophic levels, and some species of this group are sensitive indicators of changes in marine habitats [27]. Also, in estuaries, colonized by marine and freshwater organisms, changes in the structure of benthic fauna reflect environmental changes [8, 18, 28]. This applies to assessment of the role of hydrodynamic factors on sedimentation rate and resuspension of sedimented particles [29], as well as spatial changes in physico-chemical properties of water [30, 31] observed a close relationship between the rate and direction of water flow and the distribution of macrofauna. In coastal lakes, the major factors contributing to the kinetics of water particles are wind direction and speed, permanent influx of inland waters and intrusion of seawater [3, 8].

The ecological functions of the coastal lakes are affected to a great extent by the Baltic Sea properties as like salinity, temperature and water exchange [32]. The Baltic Sea is an enclosed and non-tidal ecosystem, which has steep latitudinal and vertical salinity gradients. The overall hydrography of the Baltic Sea is governed by large fresh water input in the north eastern parts that is compensated by surface water outflow through the Danish Straits and subsequent deep-water inflow, with detailed dynamics governed by meteorological forces [32]. Due to these factors the Baltic Sea is the largest brackish water body of the world with a volume of 21 000 km^2^ [33]. The Baltic Sea area constitutes such an extreme environment with a steep decrease in salinity from West (ca. 25 PSU [Practical Salinity Units]) to East (1–2 PSU). The area of the southern Baltic coast is on the way of salinity transition zone spreading from the euhaline Skagerrak and Kattegat Danish Straits to the brackish Baltic Proper, is characterised with values of about to 5–7 PSU. The strong salinity reduction from West to East decreases rapidly benthic biodiversity in the southern Baltic [34] and many species find their distribution limits over these salinity gradients [35]. Even though the Baltic is a young ecosystem, species-poor and vulnerable to the threat of invasive marine and exotic species, both the strong gradient and the rapid change of salinity conditions especially in the southern Baltic inhibit an unhindered colonisation. As a result, the Baltic benthic fauna is still largely characterized by species with obviously opportunistic life history traits [36]. The salinity reflects the variability of a series of other properties from fresh to marine waters and the complexity of the mixing zones.

The main objective of the study was to assessed to what extent a relatively small salinity gradient in coastal ecosystems determines the abundance and diversity of bottom fauna. The hypothesis was to verify that brackish and freshwater phases are alternative systems of ecological balance of coastal ecosystems, while the transitional phase is unstable. Thus, assumed that (1) a greater intrusion of seawater increases the distinctness of environmental conditions in coastal lakes; (2) a decrease in salinity gradient is associated with a decline in zoobenthic diversity and abundance; (3) the structure of benthic fauna indicates the existence of two alternative stable states and periodically appearing adaptive cycles separating them (transitional stage) due to intrusion of seawater.

## Material and methods

Permissions to carry out the study were provided by the Regional Directorate for Environmental Protection in Szczecin (no. WOPN-6401.208.2012.MS.AW and WOPN-ON.6205.30.2014.PW); Regional Directorate for Environmental Protection in Gdańsk (No. RDOŚ-Gd-PNII.6205.57.2012.KD.2 and RDOŚ-Gd.PNII.6205.11.2015.KD.1); Slowiński National Park (No. BN/P/19/2012/ii, 08/BN/2014, 04/BN/2015 and 04/BN/2016); Polish Ministry of the Environment (No. DLPpn-4102-17/964/13/RS and DZP-Wg.6401.01.39.2014.bp).

### Study area

Most of the lakes on the southern coasts of the Baltic Sea are extensive, shallow aquatic habitats with a poorly developed shoreline and remarkable fluctuations of water level (EU Habitats Directive, habitat 1150: Coastal lagoons). They are polymictic and strongly eutrophic, but they differ in morphometric, hydrological, and physico-chemical properties of water (Table 1). This causes problems with unambiguous classification of the water bodies, as they have specific features that affect their functioning.

**Table 1:** Morphometric characteristic and classification of the studies coastal lakes. Water bodies type corresponds to connectivity (water exchange with the sea) and level of salinity (Venice System, 1959). Area was measured using summer aerial photographs.

Lake	Geographic coordinates	Area (ha)	Mean depth (m)	Capacity (10^6^ m^3^)	Level of salinity	Hydrological connectivity	SB	Type of habitat
**Resko**	54°09’ N, 15°21’ E	577	2.5	7.7	β-oligohalinity	lake permanently seawater enters it by canal of the Błotnica River	257	Brackish water
**Łebsko**	54°43’ N, 17°25’ E	7020	4.7	113.5		lake permanently seawater enters it by canal of the Łeba River	276	
**Kopań**	54°29’ N, 16°27’ E	786	3.9	11.7	limnetic/ β-oligohalinity	periodically seawater lake enters it by canal	102	Transitional
**Gardno**	54°39’ N, 17°07’ E	2338	2.2	31.3		periodically seawater lake enters it by canal of the Łupawa River	165	
**Wicko Przymorskie**	54°33’ N, 16°38’ E	1058	6.1	28.5	limnetic	freshwater coastal lake isolated from sea	0	Freshwater
**Dołgie Wielkie**	54°42’N, 17°12’E	136	2.7	1.9		freshwater coastal lake, isolated from sea, surrounded by dunes	0	

SB = average number of days of seawater backflow in 2014–2015 [20].

### Sample processing

**Fig 1:**
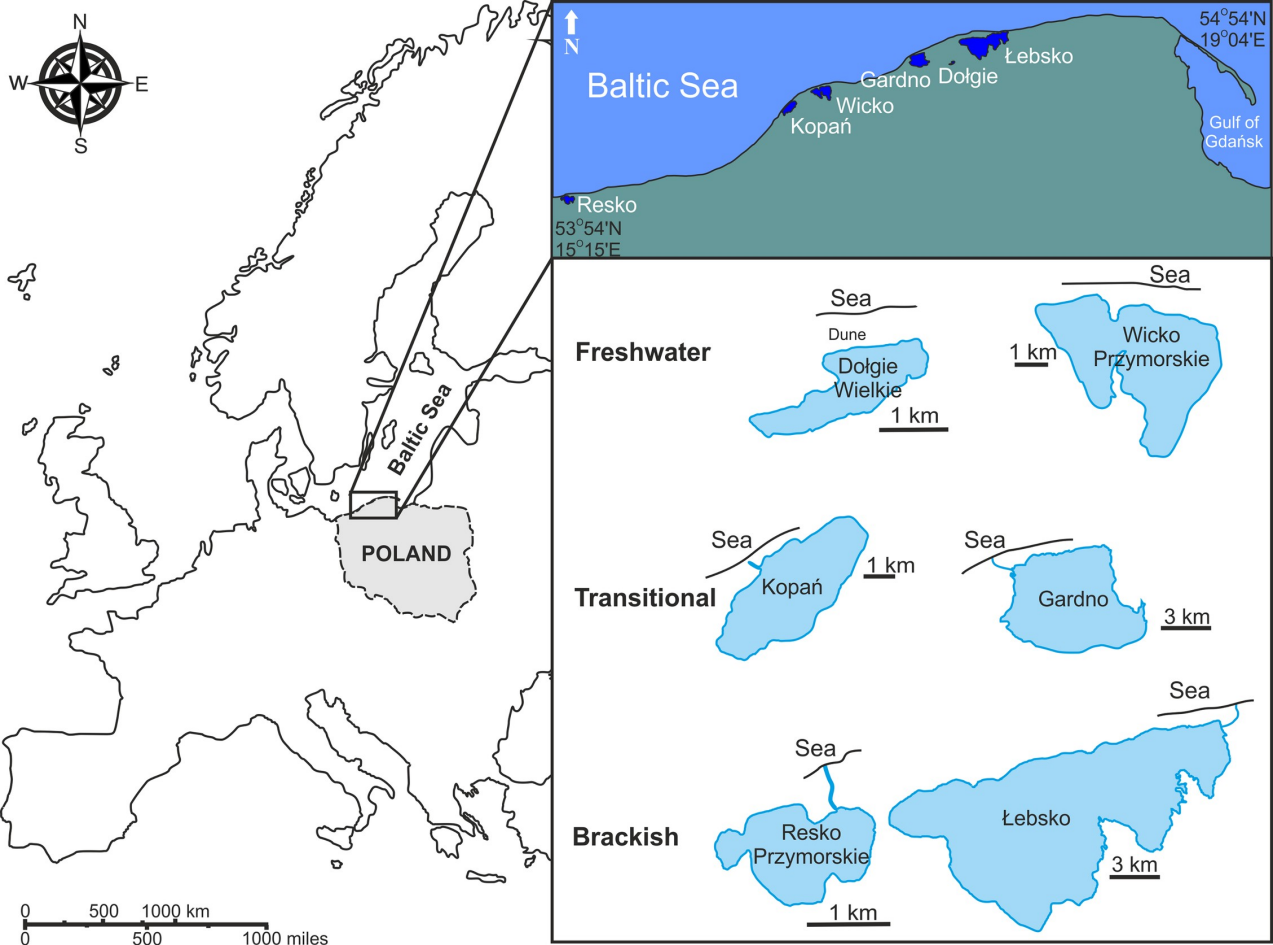
Map of the southern coast of the Baltic Sea, showing the location of the studied coastal lakes.

From six coastal water bodies (two freshwaters: Wicko Przymorskie, Dołgie Wielkie; two brackish: Łebsko, Resko; and two transitional: Gardno, Kopań, Fig 1), samples were taken every 3 months (except for winter) in 2014 and 2015. Depending on lake area, numbers of sampling stations varied from 5 on Dołgie, Kopań, Gardno and Resko, to 8 on Wicko, and 11 on Łebsko. At each site, three subsamples were collected from the bottom by using an Ekman bottom dredge with a catching area of 225 cm^2^. Solid samples were sieved through a 0.5-mm mesh and preserved with 6% formalin. Such samples were transported to a laboratory, where the collected material was sorted under a stereo microscope (Leica M60, Germany). All invertebrates were identified to the lowest possible taxonomic level and counted.

Simultaneously with biological sampling, measurements of the aquatic environment were performed. With the use of multiparameter Aquaprobe AP-7000 (Aqua Read Instrument, England), measured *in situ* electrolytic conductivity, pH, dissolved oxygen, water temperature, salinity, Chlorophyll-*a* (Chl-*a*) and total dissolved solids (TDS). Moreover, used Secchi depth (SD) to assess water transparency. The ionic composition of water samples was tested in laboratory conditions, where the cations (K^+^, Ca^2+^, Mg^2+^, Na^+^, NH_4_^+^) and anions (NO_3_^-^, NO_2_^-^, Cl^-^, SO_4_^2-^) were analysed with the use of ion chromatography (881 Compact IC Pro, Metrohm, Switzerland). Prior to analyses, samples were filtered on 0.20-μm sterile filters, and then examined with the use of Metrosep C4 250/4.0 and Metrosep A Supp 5 250/4.0 columns with Metrosep C4 Guard/4.0 and Metrosep A Supp 4/5 Guard 4.0 pre-columns, respectively. Concentrations of phosphates (TP and PO_4_^3-^) were determined in a laboratory with the use of spectrophotometry (Hach 3900, Germany) and cuvette tests (LCK349) Total organic carbon (TOC) was determined in unfiltered samples. Dissolved organic carbon (DOC) was analysed quantitatively after passing through cellulose nitrate membrane filters, with pore size 0.45 μm (Millipore). Further analysis was conducted after burning at a high temperature (Shimadzu TOC 5000 analyser, Japan) according to [37].

### Data analyses

Dispersion of sampling sites in the studied coastal lakes based on water quality parameters, similarity abundance, and α-diversity of benthic fauna were analysed with the non-metric multidimensional scaling (NMDS) ordination method using Bray-Curtis dissimilarity indices [38].

Differences between variables for lake types were tested by analysis of variance (ANOVA) with the Kruskal-Wallis test by ranks. At that stage, the data (S3 Table) were tested for normality (Shapiro-Wilk test) and homoscedasticity (Levene test), and next log (x + 1) transformed [39].

Taxa and plots were grouped based on bottom fauna community density, using two-way hierarchical agglomerative cluster analyses in PC-ORD 6.08 software [40]. Two-way cluster analysis (TWCA) independently groups samples and invertebrates, then combines them into a single diagram to allow observation of associations between groups of sample units and groups benthic fauna. The input data were analysed with the maximum variable (recommended by PC-ORD), and the matrix of variation was calculated by using Bray-Curtis distance. The matrix of agglomerative trees of hierarchical grouping was generated by using the elastic beta method (β = -0.25). Significance of differences in the multivariate structure of zoobenthic communities was tested using permutational analysis of variance [41]. The three descriptors of bottom fauna were: abundance (indiv.m^-2^), α-diversity, based on Shannon index (log_2_), and β-diversity, based on Whittaker index. Determination of differences between habitat types colonized by invertebrates (permanent categorizing factors), and lakes (random factors: two closed, two permanently open, and two periodically closed, included in the categorization) was conducted with the use of permutational (9999 replications) analysis of variance [42] conducted on a matrix of Euclidean distances. Specificity of habitats was shown on the basis of defining of indicator organisms, and determination of indicator value (IndVal) based on the abundance of *i*^th^ group of invertebrates in relation to *j*^th^ habitat type [41]. To find seasonal differences in benthic fauna abundance between lake types, we used the multi-response permutation procedure (MRPP), which is a class of multiparameter permutation tests of differences between groups. It is preferred over conventional analyses of tests based on variance because of its resistance to a lack of normal distribution of data and heterogeneous error variances [43].

At the final stage of analyses, the linear model of redundancy (RDA) was used to explain the variance in abundance of the studied groups of bottom fauna, and to indicate their relations with environmental variables and additionally with individual lake types. To assess the significance of correlations between variables and the first two axes, used the Monte Carlo test with 999 permutations. Simultaneously, selected a suitable subset of explaining variables (using a method for elimination of the factors that are not significant for the model), which represent relations between environmental variables and benthic invertebrate groups. Interactions between the abundance of benthic fauna and grouped environmental variables were explored using the Bio-Env procedure [44]. It allowed us to identify the group of variables that can explain best the changes in structure of animal communities.

## Results

### Environmental conditions

**Fig 2:**
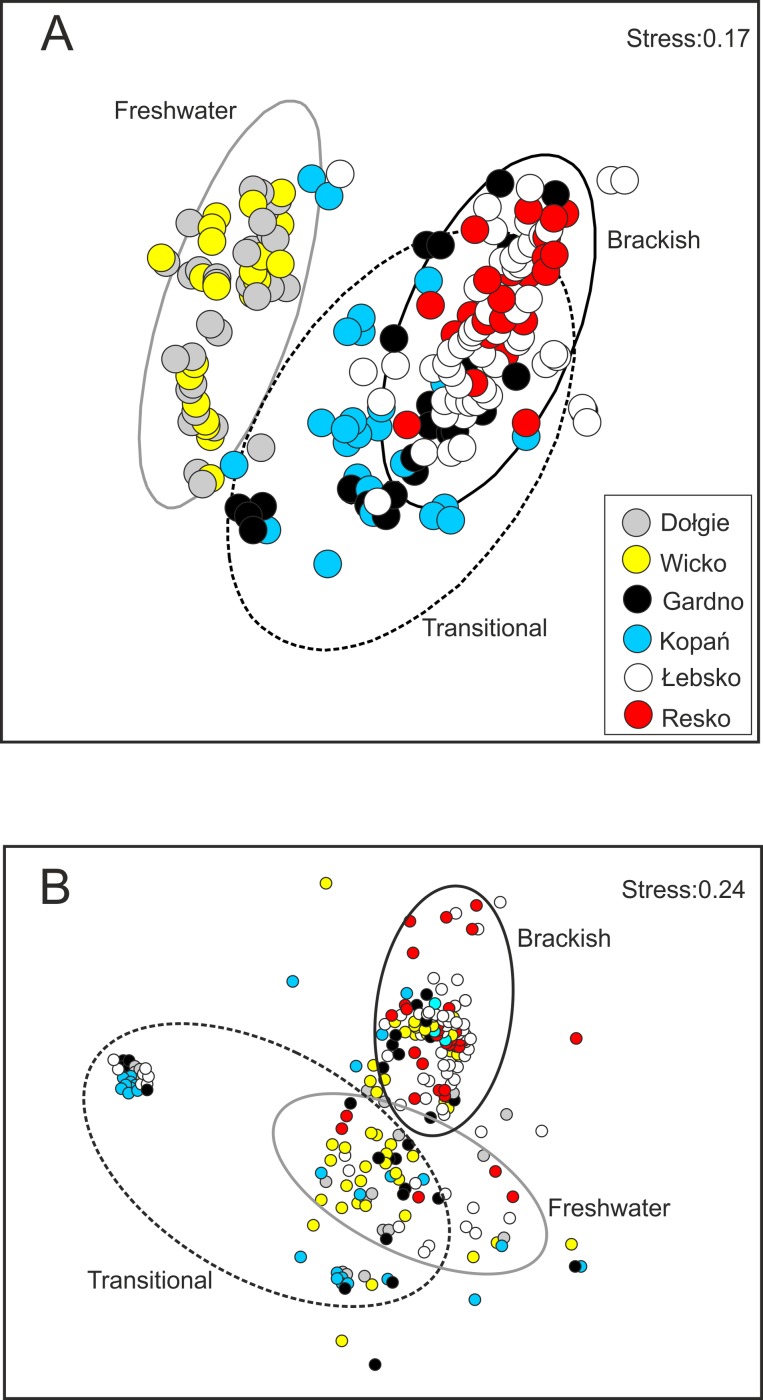
**Results of non-metric multidimensional scaling (NMDS) ordinations showing the similarity of sampling sites in the studied coastal lakes**: (A) based on water quality parameters; and (B) based on benthic macroinvertebrates structure (abundance and α-diversity).

Non-metric multidimensional scaling (NMDS) revealed remarkable differences in environmental conditions, mostly in salinity and concentrations of chlorides and sodium, which resulted from the influx of seawater to the coastal lakes (Fig 2A). The division of habitats was consistent with the Venice System for the Classification of Marine Waters, based on salinity, so below used the terms: freshwater (F), transitional (T), and brackish (B). The two axes explained as many as 85% of the variance.

The mean values of the analysed physico-chemical parameters of waters of each type of the coastal lakes are shown in Table 2. Mean Secchi depth in the studied lakes was about 0.3 m and it increased with salinity, in contrast to water temperature. Water pH in the three lake types was slightly alkaline, but the lowest values were recorded in brackish lakes and the highest in transitional ones. Fluctuations of conductivity and TDS were much larger, as they were about 25-fold higher in brackish than in freshwater lakes. Oxygen content in all lakes slightly exceeded saturation, except for slightly lower values in brackish habitats. Chl-*a* content was nearly 3-fold higher in freshwater than in brackish water bodies. P and ammonium N concentrations were the highest in freshwater lakes, while nitrate and nitrite N, in brackish ones. Organic C content (TOC and DOC) was higher in freshwater lakes than in those with periodical or permanent intrusion of seawater. Salinity reflected the level of water exchange with the sea, so it was nearly 30-fold higher in brackish than in freshwater lakes. The same applies to concentrations of all cations, as well as sulphates and chlorides, which were 3–34-fold and 21–36-fold higher in brackish than in freshwater lakes. The environmental variables (excluding mean value of Chl-*a*) showed statistically significant differences in concentrations between the analysed habitat lake types.

**Table 2:** Mean values of water parameters (± standard deviation) of coastal lakes in 2014–2015 (n = 232) and results of one-way ANOVA evaluating differences in results.

	Unit	Freshwater n = 78	Transitional n = 59	Brackish n = 95	*p*
**SD**	m	0.27 (0.09)	0.33 (0.14)	0.35 (0.11)	0.001
**Temp.**	^o^C	16.5 (4.7)	16.3 (4.2)	15.9 (4.1)	0.0003
**pH**	-	8.70 (0.37)	8.83 (0.43)	8.51 (0.36)	0.001
**DO**	%	103.9 (19.8)	108.3 (21.9)	93.6 (21.6)	0.001
**EC**	μS cm^-1^	230 (134)	2556 (2237)	5521 (2429)	0.0001
**TDS**	mg L^-1^	137 (1)	1628 (40)	3415 (43)	0.0001
**Salinity**	PSU	0.10 (0.07)	1.28 (1.06)	3.07 (1.61)	< 0.0001
**Chl-*a***	μg L^-1^	55.9 (100.5)	22.8 (20.4)	20.1 (32.2)	0.053
**NO**_**2**_^**-**^	mg L^-1^	0.043 (0.028)	0.058 (0.023)	0.067 (0.080)	0.001
**NO**_**3**_^**-**^	mg L^-1^	0.735 (0.408)	0.991 (0.748)	0.998 (0.634)	0.004
**NH**_**4**_^**+**^	mg L^-1^	0.373 (0.574)	0.304 (0.318)	0.245 (0.224)	0.005
**PO**_**4**_^**3-**^	mg L^-1^	0.237 (0.105)	0.192 (0.100)	0.192 (0.106)	0.002
**TP**	mg L^-1^	0.669 (0.386)	0.462 (0.274)	0.365 (0.456)	0.0001
**TOC**	mg L^-1^	23.4 (14.9)	18.8 (14.0)	13.8 (4.7)	0.001
**DOC**	mg L^-1^	13.2 (5.1)	10.0 (6.5)	7.8 (3.4)	0.001
**Cl**^**-**^	mg L^-1^	37.7 (20.7)	716.6 (539.4)	1381.1 (0.495)	< 0.0001
**SO**_**4**_^**2-**^	mg L^-1^	10.8 (5.9)	108.5 (71.5)	229.2 (92.1)	0.0001
**Li**^**+**^	mg L^-1^	0.010 (0.005)	0.018 (0.008)	0.036 (0.038)	0.0002
**Na**^**+**^	mg L^-1^	24.2 (14.4)	429.4 (286.7)	822.7 (382.1)	< 0.0001
**K**^**+**^	mg L^-1^	3.3 (1.8)	16.1 (11.2)	29.7 (12.5)	0.0004
**Ca**^**2+**^	mg L^-1^	17.4 (13.0)	35.4 (12.9)	56.2 (19.7)	0.0002
**Mg**^**2+**^	mg L^-1^	4.3 (6.8)	49.2 (32.6)	89.4 (43.4)	0.0003

SD = Secchi depth; TDS = total dissolved solids; EC = conductivity; DO = dissolved oxygen; TP = total phosphorus; TOC = total organic carbon; DOC = dissolved organic carbon.

*p* values modified by the Bonferroni procedure for multiple comparisons show no significant effect.

### Salinity level and assemblage similarities

The differences in abundance and structure of macrobenthic fauna observed between the identified types of hydrological connectivity are presented in S1 Table. A total of 45,330 benthic fauna representatives belonging to 28 species, 14 genera, 1 family and 1 subdivision were identified.

The scaling method NMDS revealed that the distribution of biological data was not normal for individual lakes, considering the level of their salinity. It allowed us to determine the level of dispersion of data from individual lakes. The NMDS (Fig 2B) shows a relatively low gradient of dispersion of sampling sites (stress = 0.24) as a response of invertebrates to the influence of brackish water from the Baltic Sea. Nevertheless, spatial differentiation of benthic community structure was more evident in sampling sites of transitional lakes, than in brackish and freshwater lakes (Fig 2B). All the analysed descriptors significantly differed between the types of coastal lakes (Table 3). The abundance of benthic macrofauna was the highest in brackish lakes and the lowest in transitional ones, which was also reflected in their species α-diversity.

**Table 3:** Mean density (indiv. m^-2^ ± standard deviation) and percentage contributions of groups of invertebrates in coastal lakes of the southern Baltic Sea and results of non-parametric Kruskal-Wallis test.

	Freshwater n = 78		Transitional n = 59		Brackish n = 95		*p*
**Species richness**	20	%	21	%	35	%	< 0.0001
**Total density**	484.7 (1058.9)		245.4 (445.0)		937.6 (938.0)		< 0.0001
**α-diversity**	0.586 (0.402)		0.467 (0.501)		0.756 (0.471)		0.001
**Oligochaeta**	116.4 (225.7)	24.0	31.1 (52.6)	12.7	236.4 (354.1)	25.2	< 0.0001
**Polychaeta**	0.0		0.0		11.4 (76.2)	1.2	0.11
**Crustacea**	0.0		6.2 (21.5)	2.5	275.8 (354.1)	29.4	< 0.0001
**Hirudinea**	0.0		0.7 (5.7)	0.3	1.2 (9.3)	0.1	0.29
**Diptera larvae**	362.6 (1023.2)	74.8	203.2 (420.3)	82.8	409.2 (454.1)	43.6	< 0.0001
**Hemiptera**	0.0		0.0		0.3 (2.1)	+	0.24
**Trichoptera larvae**	0.4 (2.4)	0.1	0.5 (3.8)	0.2	0.2 (1.5)	+	0.75
**Ephemeroptera larvae**	0.2 (1.7)	+	0.0		0.3 (2.1)	+	0.53
**Gastropoda**	2.3 (17.0)	0.4	2.5 (11.3)	1.0	2.5 (11.1)	0.3	0.61
**Bivalvia**	2.8 (16.8)	0.6	1.2 (4.9)	0.5	0.3 (2.1)	+	0.36

+ <0.1%

Three habitat types were dominated by the Diptera (chironomid larvae) and the Oligochaeta, and their summed percentage contribution to the total abundance decreased with rising salinity from 98.8% to 68.8% (Table 3). The relative density or the percentage contributions of each group in different habitat types indicate a higher relevance of Diptera in transitional waters and a lower importance of this group in brackish waters. Although the density of Diptera is higher in brackish habitats, some other groups also reach higher densities there (e.g Oligochaeta and Crustacea). A similar trend was observed for Bivalvia, but the differences were smaller and less significant. Rising salinity was associated with significantly increasing contributions of Crustacea as well as Polychaeta and Hemiptera, but without significant differences between lake types (Table 3). Only in transitional lakes Trichoptera larvae, Hirudinea, and Gastropoda had the highest contributions to the total density of benthic fauna.

Among the studied invertebrate groups in relation to habitat types, performed an indicator value (IndVal) analysis, which generally showed preference for brackish water, except for the Trichoptera (represented mainly by *Economus tenellus*), which flourished in freshwater lakes (Table 4, S2 Table).

**Table 4:** Results of assessment based on the Indicator Value (IndVal) method for habitat types.

Name of taxa	Max. habitat	Observed IndVal	IndVal from randomized groups		*p*(MC)
			Mean	S.Dev.	
**Oligochaeta**	Brackish	59.9	38.2	3.18	0.0002
**Polychaeta**	Brackish	6.7	6.7	3.13	0.323
**Crustacea**	Brackish	74.1	18.8	4.93	0.0002
**Hirudinea**	Brackish	4.9	4.4	2.33	0.543
**Diptera**	Brackish	46.3	38.3	3.69	0.034
**Hemiptera**	Brackish	3.3	3.3	0.05	1.000
**Trichoptera**	Freshwater	2.7	5.0	2.39	1.000
**Ephemeroptera**	Brackish	4.4	4.2	2.39	0.764
**Gastropoda**	Brackish	6.7	7.4	3.11	0.542
**Bivalvia**	Brackish	5.6	9.6	5.17	0.651
Averages		15.6	13.6	3.04	0.051

Denotations: *p*(MC)—proportion of randomized trials with indicator value equal to or exceeding the observed indicator value. p = (1 + number of runs > = observed)/(1 + number of randomized runs). Max. habitat = Habitat identifier for group with maximum observed IndVal. Randomization test for sum of IndVal observed sum of IndVal across all variable = 505.4; number of randomization runs with sum of IndVal ≥ observed value = 0; *p* = 0.0002

**Fig 3:**
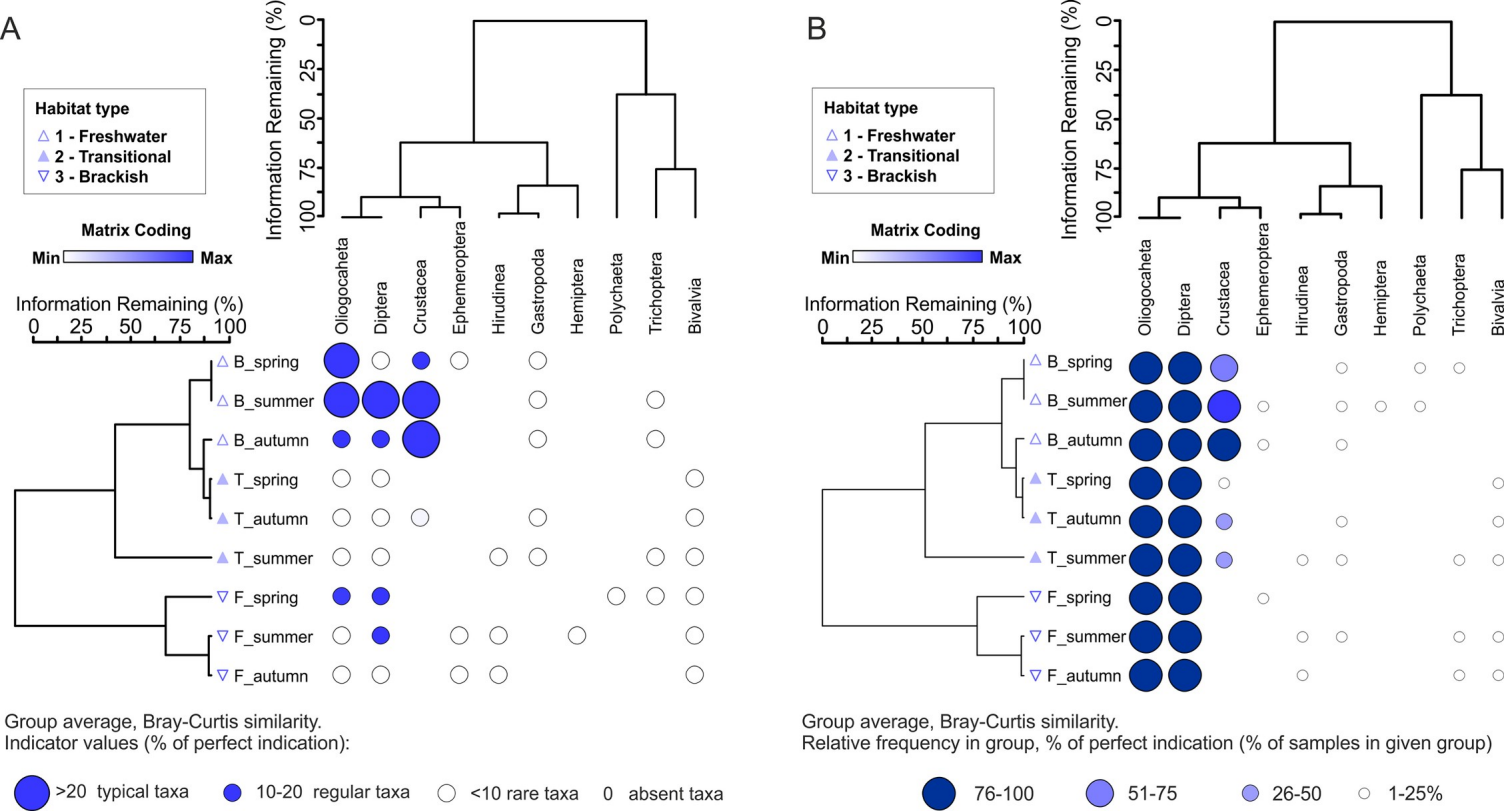
Two-way cluster analysis (TWCA) based on the relative value of biotic variables in samples from three types of coastal lakes (F = Freshwater; T = Transitional; B = Brackish) in different seasons. The horizontal dendrogram groups invertebrates and the main representatives according to similarity. The vertical dendrogram groups samples according to indicator values of invertebrates (A) and relative frequency in the group (B). Cold map colours indicate minimum (white) to maximum (blue) value of zoobenthos groups, wherein each element is scaled to its maximum contribution to the total recorded value in a lake type.

TWCA (Fig 3) at the level of seasonal analyses confirmed the coexistence of three lake types, generating separate clusters. Only results from spring and summer in transitional lakes are similar to brackish lakes. Among the analysed members of benthic fauna, the Oligochaeta were typical of brackish habitats in spring and summer, and additionally larvae of Diptera in summer (Fig 3A). No taxa were typical of transitional lakes, whereas crustaceans were typical of freshwater habitats in summer and autumn. TWCA based on frequency indicated that two groups of benthic fauna were regularly observed in all seasons: the Oligochaeta and Diptera (Fig 3B). Simultaneously, crustaceans were a significant component of benthic fauna in brackish lakes, especially in autumn.

In individual seasons, densities of invertebrates differed primarily between transitional and brackish lakes (Table 5). However, the significance of differences increased during the year, reaching the highest value in autumn. In the same season, density of fauna differed significantly also between the extremes of salinity gradient (freshwater vs. brackish).

**Table 5:** Multi-response permutation procedures (MRPP) for seasonal densities of invertebrates for types of coastal lakes.

Groups (identifiers) Compared	Season	T	A	*p*
**Freshwater vs Transitional**	Spring	0.2708	-0.0097	0.48
**Freshwater vs Brackish**		-0.7067	0.0200	0.21
**Transitional vs Brackish**		-2.8704	0.0952	0.02
**Freshwater vs Transitional**	Summer	-0.0509	0.0017	0.37
**Freshwater vs Brackish**		-1.3185	0.0405	0.10
**Transitional vs Brackish**		-4.8850	0.1522	0.002
**Freshwater vs Transitional**	Autumn	0.0783	-0.0023	0.42
**Freshwater vs Brackish**		-4.1610	0.1428	0.005
**Transitional vs Brackish**		-5.3498	0.1653	0.001

Chance-corrected within-group agreement, A = 0.087

A = 1 - (observed delta/expected delta)

A_max_ = 1 when all items are identical within groups (delta = 0)

A = 0 when heterogeneity within groups equals expectation by chance

A < 0 with more heterogeneity within groups than expected by chance

PERMANOVA results based on benthic structure indices such as density and diversity indicated a differentiation between lagoon types. Testing the effects of habitat types on benthic invertebrates structure (Table 6), revealed that it was most abundant in brackish lakes, which clearly distinguished it from freshwater lakes. Results of the analyses were consistent, irrespective of whether they were considered in respect of presence/absence or relative density of benthic invertebrates. Indices of α- and β-diversity significantly differed between the lake types that differed most strongly in salinity (Table 6). In the habitats characterized with active exchange of water with the sea (Kopań and Gardno lakes), changes in structure and density of invertebrates were dependent on the presence or absence of connection with the sea. In periods of lack of connection with the sea, in the so-called limnic phase, benthic invertebrates structure significantly differed from the oligohaline phase (i.e. associated with the influx of brackish water) and was characterized by greater abundance.

**Table 6:** Permutational analysis of variance (PERMANOVA) results, testing the effects of lake types (Freshwater, Transitional and Brackish) and lakes (two of each type) on the species richness, density and diversity of invertebrates.

	Sources of variation	df	SS	MS	pseudo F values	*p*(MC)
**Species richness**	Habitat type	2	0.7661	0.3830	2.7080	0.0001
	Lakes	5	1.4729	0.2946	2.0828	0.0001
	Residual	72	10.184	0.1415		
Pair-wise tests						
Compared	Freshwater vs Transitional				83.84	0.0001
	Transitional vs Brackish				96.33	0.0001
	Freshwater vs Brackish				198.60	0.0001
**Density**	Habitat type	2	2.7601	1.3801	2.5033	0.0001
	Lakes	5	4.5475	0.9095	1.6498	0.0001
	Residual	72	39.694	0.5513		
Pair-wise tests						
Compared	Freshwater vs Transitional				2.75	0.06
	Transitional vs Brackish				18.10	0.0001
	Freshwater vs Brackish				7.66	0.0004
**Total α-diversity**	Habitat type	2	0.8813	0.4407	1.4174	0.0003
	Lakes	5	2.2804	0.4561	1.4670	0.0001
	Residual	72	22.385	0.3109		
Pair-wise tests						
Compared	Freshwater vs Transitional				206.80	0.0001
	Transitional vs Brackish				243.40	0.0001
	Freshwater vs Brackish				482.20	0.0001
**Total β-diversity**	Habitat type	2	0.0098	0.0049	0.0781	0.81
	Lakes	5	0.2205	0.0441	0.7063	0.03
	Residual	72	4.496	0.0624		
Pair-wise tests						
Compared	Freshwater vs Transitional				264.60	0.0001
	Transitional vs Brackish				320.80	0.0001
	Freshwater vs Brackish				669.90	0.0001

Analysis performed based on Bray-Curtis dissimilarity indices. *p*(MC): *p*-value obtained with Monte Carlo permutation test. Bold values indicate significant differences at *p*<0.05.

### Environmental variables and zoobenthic communities

Out of the 22 environmental variables analysed initially in the RDA model, focused on eight physico-chemical parameters, which markedly affected the quality of the model (Table 7). Consequently, the final model did not take into account one physical variable (Secchi depth) and 13 chemical ones (pH, K^+^, Li^+^, Mg^2+^, Cl^-^, DOC, TOC, TDS, Chl-*a*, PO_4_^3-^, NH_4_^+^, SO_4_^2-^ and NO_2_^-^) as redundant or only slightly improving the quality of the constructed model.

**Table 7:** The explanatory variables selected that represent a significant relationship between the groups (marginal and conditional effects).

Variables	Marginal Effects	Conditional Effects		
	λ1	λA	*p*	F-ratio
**EC**	0.23	0.23	0.002	70.03
**Salinity**	0.21	0.04	0.002	52.31
**Temperature**	0.21	0.01	0.010	36.08
**Na**^**+**^	0.20	0.04	0.004	29.16
**NO**_**3**_^**-**^	0.20	0.01	0.022	11.75
**Ca**^**2+**^	0.19	0.02	0.011	28.61
**TP**	0.03	0.01	0.014	27.09
**DO%**	0.03	0.01	0.006	26.76

Lambda denotes the amount of variability in the groups data that would be explained by a constrained ordination model using that variable as the only explanatory variable. Variables not used in the table were statistically insignificant.

**Fig 4:**
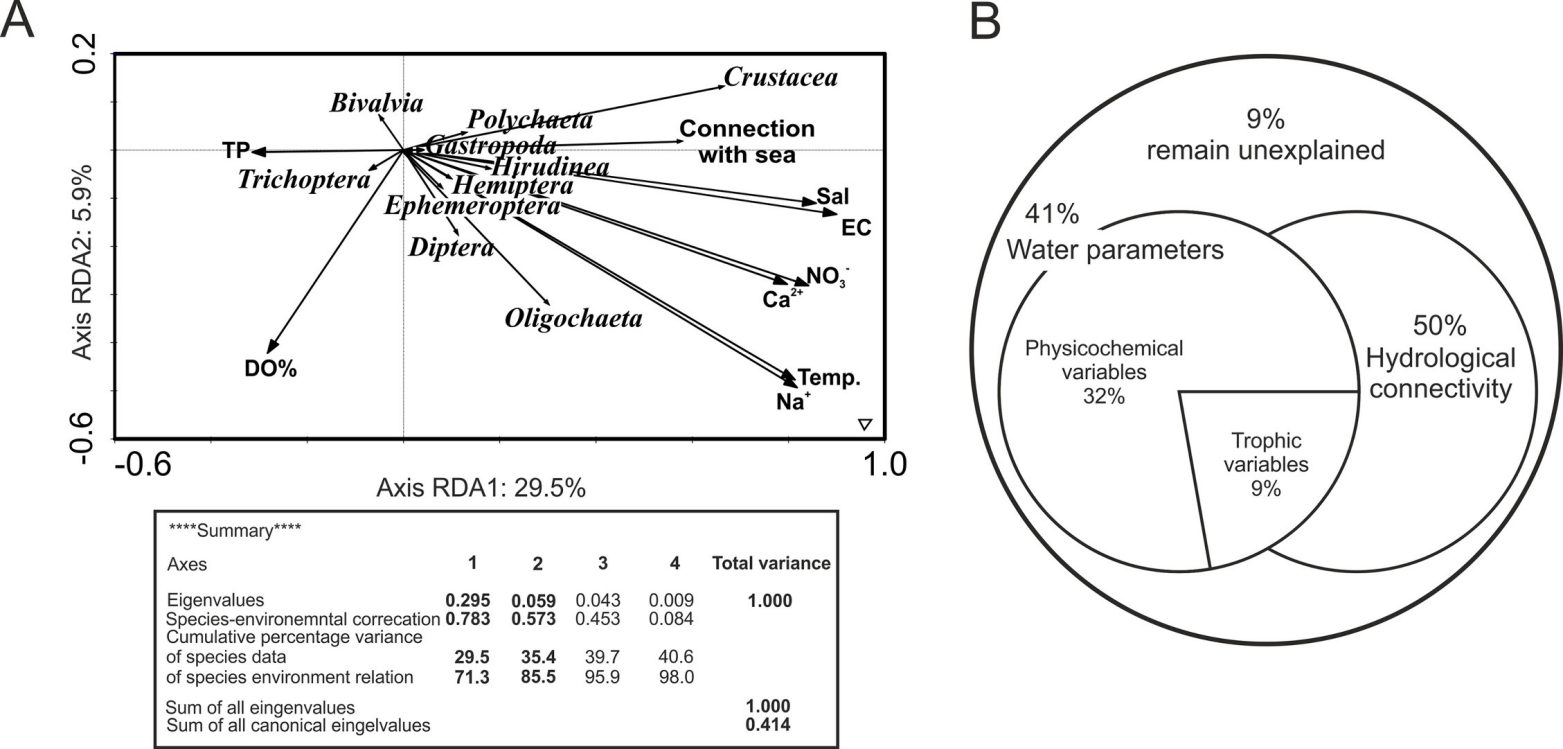
**Results of redundancy analysis (RDA)**: (A) a biplot of significant environmental variables and zoobenthos density (*p* < 0.05); (B) unique and shared fractions (Bio-Env procedure) of the total variation of zoobenthos composition, explained by hydrological connection with the sea and the contribution of physico-chemical and trophic parameters of water.

In the generated RDA model, the first axis explained 29.5% of the variance, while the second axis explained 5.9% of the variance (in total 35% of the total inconsistency) in abundance of invertebrates, and all the canonical axes were significant (Monte Carlo test, *p* = 0.002). The first factor was strongly linked with conductivity and concentrations of ions (related to salinity), while the second one with hydrological conditions (hydrological connectivity) (Fig 4A).

Bio-Env analysis (Fig 4B) showed that the categorized environmental conditions (based on lake type, Table 1) were the best correlated with invertebrate community composition and explained 50% of the variance in their density, whereas physico-chemical variables, including temperature, conductivity, Na^+^, Ca^2+^, Mg^2+^, SO_4_^2-^, DO and salinity) explained 32% of the variance in density of invertebrates. Trophic variables (NO_3_^-^, TP) explained 9% of the variance in benthic invertebrates structure. The total variance of the three groups of variables accounted for 91% of the variance in abundance of invertebrates, while only 9% remained unexplained.

**Fig 5:**
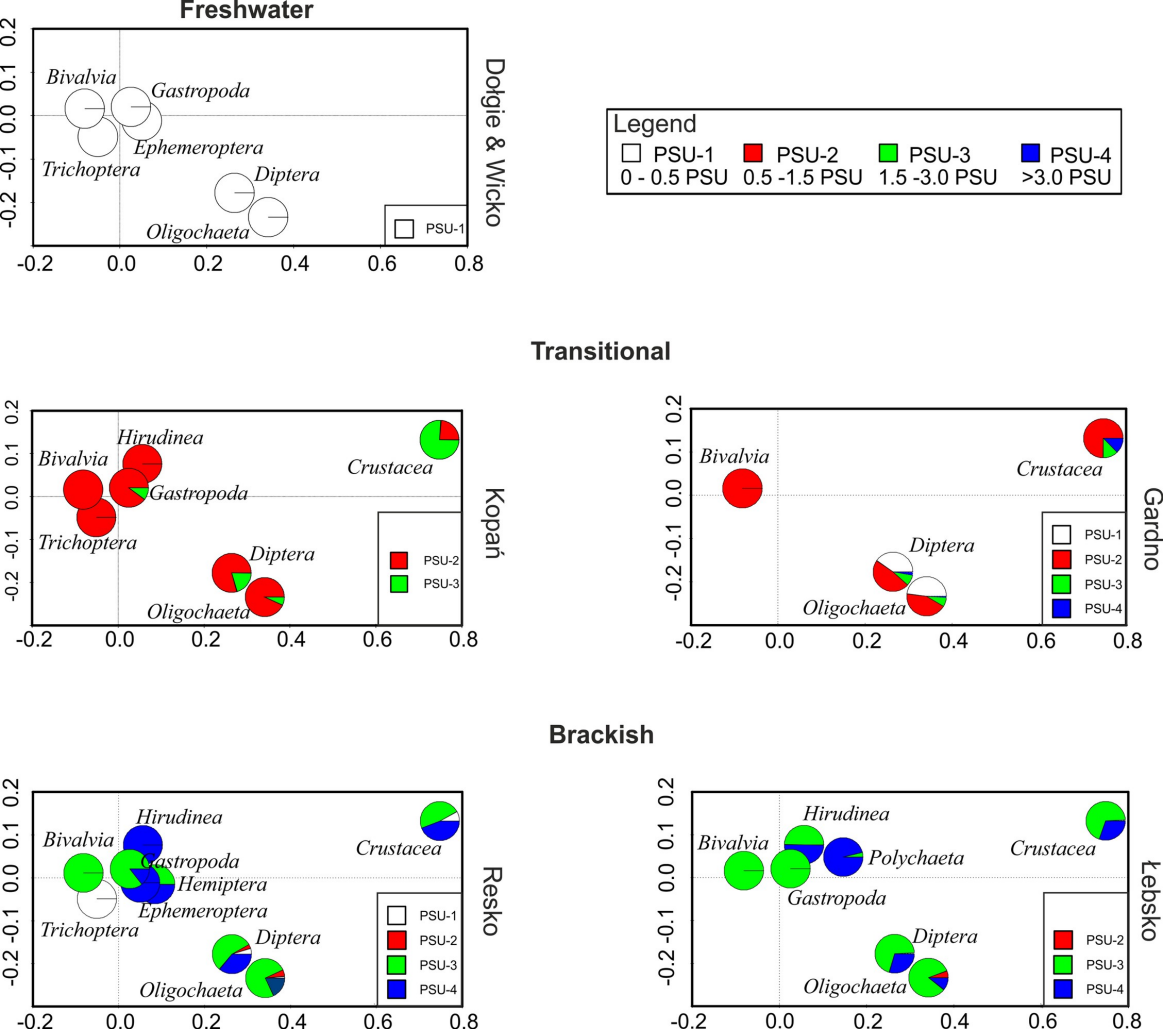
Results of redundancy analysis (RDA) performed on percentage contributions of zoobenthos groups for salinity level in types of coastal lakes (*p* < 0.05).

RDA also showed preferences of invertebrates for habitats with various levels of salinity (Fig 5). Generally, taxonomic diversity increased with increasing salinity. When salinity level was low, with small fluctuations (= freshwater lake), 60% of the recorded groups of benthic invertebrates appeared. The structure of fauna did not differ among coastal lakes of this type. The variation of transitional lakes was much greater, as they shared four groups, but in Lake Kopań, with the lowest salinity, two additional groups appeared (Gastropoda, Trichoptera). Benthic animals colonizing this type of lakes preferred low salinity (0.5–1.5 practical salinity units, PSU), and the highest salinity favoured the appearance of crustaceans. In brackish lakes, the diversity of benthic invertebrates reached the maximum values, slightly higher in Resko than in Łebsko. In lakes of this type, fauna preferred habitats with moderate salinity (1.5–3.0 PSU), and only polychaetes (*Mysis mixta*, *Hediste diversicolor* or *Pygospio elegans*) and Hirudinea (*Pisicola geometra*) appeared when salinity was high (>3.0 PSU; Fig 5, S1 Table).

## Discussion

Understanding of the mutual relationships between the composition and distribution of biological communities and changing environmental conditions at the local or regional level is one of the major challenges of ecological research [28]. Partitioning of diversity measures enables us to understand better the mechanisms that shape the structure of communities along environmental gradients [8], such as salinity in coastal lakes [45]. It acts like an environmental filter for macroinvertebrates, preserving only the species whose functional or phenotypic traits have resulted in a wide ecological tolerance [8, 34]. Otherwise, after migration to areas with variable salinity, regulation of internal ion concentrations would lead to energy exhaustion, and individuals could limit reproduction or even die because of a lack of suitable morphological and/or anatomic adaptations [45]. The environmental change that happen in ICOLLs (phase open or close) can affect colonization opportunity by invertebrates [19, 25]. Animals can reach the lagoons but eventually are not able to survive there due to the unfavourable environmental conditions [18]. This applies mainly to marine animals migrating to the freshwater coastal lakes (S1 Table). This problem in the lagoons studied seems to be insignificant since the salinity range is very low. In addition, when the lagoon is open to the surrounding water it might not be coincident with the immigration phase of most animals. This applies particularly to water bodies with a noticeable salinity gradient, e.g. the Baltic Sea [32, 46] or moderately saline estuaries [47].

Our study supplements those reports with data on changes in groups of macrozoobenthos in Baltic coastal lakes with a small gradient of salinity (0.5–3.0 PSU), resulting from varying degrees of connection with the sea, which is brackish (~7.0 PSU). Generally, freshwater communities of invertebrates are poorer than those of brackish and transitional ecosystems, but this does not affect the dominance structure shaped mostly by the Oligochaeta and Diptera (chironomid larvae), and in brackish waters also the Crustacea [18, 48]. The greater diversity of benthic fauna in brackish waters, as compared to freshwater habitats, reflects both the contribution of marine organisms (e.g. *Pygospio elegans*, *Hediste diversicolor*, *Gammarus oceanicus*, *Corophium volutator* or *Idotea balthica*), and the presence of a strong environmental gradient, which determines habitat heterogeneity [49].

Our findings are consistent with other reports, showing a linear relationship between species richness and salinity [46, 47, 50, 51]. This decreasing trend in species richness is explained by the theory proposed by [52], who studied the distribution of communities along a salinity gradient in the Baltic Sea. According to her theory of macrozoobenthic diversity patterns along the gradient, the community of macroinvertebrates reached the highest species richness in the area with the highest salinity, and it declined with decreasing salinity, reaching a minimum in the area labelled “Artenminimum”, where salinity ranged from 5 to 8 PSU. Species richness increased again with a greater influx of freshwater and appearance of species from freshwater habitats. In waters of shallow lagoons with regular intrusion, the abundance of bottom fauna characteristic of benthos and periphyton (if plants are abundant) may be high and such “refreshing” is thus potentially important, as it creates so-called „windows of opportunity” [53, 54].

When attempting to determine the status of lakes on the basis of their evolution in time, noted that brackish and freshwater lakes can be treated as contrasting alternative phases of ecological balance, whereas transitional lakes are intermediate between them. Changes in salinity regime result in a flow of resources and habitat heterogeneity (physico-chemical gradient), which directly influences the regional species diversity, abundance, and trophic state. When there are no barriers to free migration of animals, values of predictors describing benthic fauna increase, whereas isolation leads to simplification of its composition, with species loss and decrease in α-diversity (Table 3). Transitional lakes are particularly important for understanding the effects of intrusion of saline water on biological and physico-chemical parameters between brackish and freshwater lakes. During short-term shifts in salinity of transitional lakes, remarkable environmental changes take place [19, 31, 55, 56] along the gradient between the stable freshwater a brackish phase. Transitional lakes resemble oceanic intermittently closed/open lagoons and lakes (ICOLLs), and are characterized by periodical influx of seawater, which leads to high internal variation of habitats between seasons and years [57]. The possible explanation of the structure of benthic invertebrate communities of the transitional state reflects an intermediate pattern of salinity level, as compared to the stable brackish and freshwater phases. Periodical influx of seawater is a kind of “fuel” that initiates changes according to the assumptions of adaptation cycles [58].

Intrusion of seawater cause rapid changes, which can be divided into several stages: growth and development, stabilization, creative destruction, and reorganization. As a result of such a cycle, new species of benthic fauna can appear in transitional lakes. When time without intrusion of seawater is prolonged, the structure of macrozoobenthos changes, leading to a species diversity decline and modification of species composition (Table 1, S1 Table). A similar consequence, however, in the short-term perspective causes the inflow of sea waters [8, 19, 21, 49, 59]. Intrusion of seawater can be accelerated by storms on the Baltic Sea, which are sudden and violent, with wind speed reaching 60–90 km/h (occasionally up to 130 km/h) and usually last only one day. This applies particularly to autumn, winter and spring, but in the 21^st^ century the frequency of storms increases [60]. During storms, wave height reaches 3–5 m, but sometimes up to 12 m, and sea level may increase within a few hours to 1.0–1.5 m above the standard level. As a result, brackish water enters river mouths and coastal lakes that are permanently or periodically connected with the sea. In the latter case, our results confirm that even occasional intrusion of seawater initiates adaptive cycles, resulting in a relatively low diversity and abundance, as compared to lakes that are permanently isolated or open.

Natural and gradual transition from the brackish to freshwater phase is a long-term process, which results from large-scale phenomena (e.g. changes in sea level and climate) as well as local ones (e.g. sedimentation along shores, drift, shoreline morphology) [30, 61]. The impoverishment of benthic macrofauna and replacement of marine species by freshwater ones also contribute to changes in trophic status of water bodies and remarkable declines in habitat heterogeneity.

The Baltic Sea and its catchment are one of the most intensively studied marine regions in the world, and some continuous datasets date back from the 1950s. Nevertheless, there are still some knowledge gaps, so in this work studied the mechanisms of functioning of ecosystems associated with sea activity. The results indicate that the structure of communities of benthic macroinvertebrates in Baltic coast lakes are strongly linked with their salinity, which acts as an environmental stressor in respect of species diversity eliminating stenohaline species. The gradient results in maintenance of two distinct, relatively stable phases of ecological balance. Intrusion of seawater to transitional lakes forces the benthic fauna to adapt to new conditions, leading to a decline in their abundance and α-diversity, as compared to areas with stable low or high salinity.

## Supporting information

S1 Table Composition and mean abundances of macroinvertebrates (indiv m-2 ± standard deviation) at the coastal lakes of the Baltic Sea. (DOCX)

S2 Table Results of assessment based on the Indicator Value (IndVal) method for habitat types vs. all taxa, S.D.–standard deviation. (DOCX)

S3 Table The database used in the statistical analyses. (XLSX)
