# Supplementary material for: Patterns of salinity regime in coastal lakes based on structure of benthic invertebrates
Source: PLoS One. 2018 Nov 26;13(11):e0207825. doi: 10.1371/journal.pone.0207825 (PMC6257944; doi:10.1371/journal.pone.0207825)
Supplement: S1 Table — (DOCX) [file pone.0207825.s001.docx]

**S1**. **Composition and mean abundances of macroinvertebrates (indiv m^-2^ ± standard deviation) at the coastal lakes of the Baltic Sea.**

|  | **Freshwater** | | **Transitional** | | **Backish** | |
| --- | --- | --- | --- | --- | --- | --- |
|  | **Dołgie Wielkie**  **n=29** | **Wicko**  **n=47** | **Kopań**  **n=29** | **Gardno**  **n=30** | **Resko**  **n=29** | **Łebsko**  **n=66** |
| Oligochaeta | 133.9 ±  159.5 | 105.5 ± 258.5 | 14.8 ±  2.9 | 47.4 ±  66.0 | 341.7±  475.6 | 188.6 ± 290.4 |
| *Hediste diversicolor* | 0 | 0 | 0 | 0 | 0 | 3.1 ±13.2 |
| *Pygospio elegans* | 0 | 0 | 0 | 0 | 0 | 13.5 ±79.2 |
| *Mysis mixta* | 0 | 0 | 0 | 0 | 0 | 0.9 ±6.6 |
| *Asellus aquaticus* | 0 | 0 | 0 | 0 | 0.5 ±2.9 | 1.8 ±13.2 |
| *Gammarus debeni* | 0 | 0 | 7.9 ±23.2 | 4.0 ±18.1 | 60.2 ±185.6 | 10.5 ±26.4 |
| *Gammarus* *oceanicus* | 0 | 0 | 0.5 ±2.9 | 0 | 6.7 ±31.9 | 1.1 ±6.6 |
| *Corophium volutator* | 0 | 0 | 0 | 0 | 3.0 ±46.4 | 0 |
| *Idotea balthica* | 0 | 0 | 0 | 0 | 1.5 ±8.7 | 0 |
| *Neomysis integer* | 0 | 0 | 0 | 0 | 0 | 3.1 ±6.6 |
| *Glossiphonia complanata* | 0 | 0 | 0.5 ±2.9 | 0 | 0 | 0 |
| *Erpobdella octoculata* | 0 | 0 | 1.0 ±5.8 | 0 | 3.0 ±17.4 | 0 |
| *Pisicola geometra* | 0 | 0 | 0 | 0 | 0 | 0.4 ±5.2 |
| *Chironomus* *plumosus* | 126.4 ±  365.4 | 80.9 ±  145.7 | 7.9 ±  84.1 | 163.9 ± 369.4 | 450.4 ± 478.5 | 278.8 ± 389.4 |
| Chironomidae n.det. | 0.5 ±2.9 | 2.2 ±9.4 | 0 | 4.4 ±12.2 | 4.9 ±11.6 | 5.4 ±13.2 |
| *Dicrochironomus* sp. | 0 | 0.6 ±4.7 | 0 | 0 | 0.5 ±2.9 | 2.5 ±13.2 |
| *Procladius* sp. | 13.3 ±31.9 | 11.7 ±258.5 | 2.5 ±8.7 | 41.0 ±32.3 | 8.4 ±26.1 | 8.1 ±33.0 |
| *Polypedilum* sp. | 96.8 ±  356.7 | 242.3 ± 1222.0 | 131.4 ± 466.9 | 16.3 ±  51.7 | 41.0 ±  95.7 | 5.9 ±  297.0 |
| *Psectrocladius* sp. | 1.0 ±2.9 | 6.5 ±42.3 | 2.0 ±8.7 | 7.9 ±27.4 | 3.9 ±31.9 | 0.7 ±4.5 |
| *Micotendipens* sp. | 0 | 0 | 0 | 0 | 0 | 1.1 ±6.6 |
| *Einfeldia* sp. | 0 | 2.8 ±18.8 | 0 | 0 | 7.9 ±43.5 | 9.0 ±46.2 |
| *Clunio* sp*.* | 0 | 0 | 0 | 0 | 0 | 0.4 ±3.1 |
| *Pelopia* sp*.* | 0 | 2.5 ±18.8 | 0.5 ±2.9 | 0 | 0 | 0.2 ±2.4 |
| *Sergentia* sp. | 48.9 ±113.1 | 36.7 ±94.0 | 1.0 ±5.8 | 24.2 ±48.6 | 5.4 ±20.3 | 26.7 ±85.8 |
| *Bezzia* sp. | 8.9 ±26.1 | 5.2 ±159.8 | 0 | 2.5 ±6.2 | 30.1 ±136.3 | 6.7 ±13.2 |
| *Diamesa campestris* | 0 | 0.3 ±0.5 | 0 | 0.5 ±3.4 | 0 | 0 |
| *Tabanus* sp. | 0 | 0 | 0.5 ±2.9 | 0 | 0 | 0 |
| *Chaoborus* sp*.* | 1.0 ±5.8 | 0 | 0 | 0 | 0 | 0 |
| *Corixa* sp. | 0 | 0 | 0 | 0 | 1.0 ±2.9 | 0 |
| *Ecnomus tenellus* | 0.5 ±2.9 | 0 | 0 | 0 | 0 | 0 |
| *Limnephilus* sp. | 0.5 ±2.9 | 0 | 1.0 ±5.8 | 0 | 0.5 ±2.9 | 0 |
| *Caenis macrura* | 0.5 ±2.9 | 0 | 0 | 0 | 0 | 0 |
| *Acentria ephemerella* | 0 | 0 | 0 | 0 | 0.5 ±2.9 | 0 |
| *Cataclysta lemnata* | 0 | 0 | 0 | 0 | 0.5 ±2.9 | 0 |
| *Bithynia tentaculata* | 0 | 0 | 2.0 ±11.6 | 0 | 3.0 ±17.4 | 0.4 ±6.6 |
| *Valvata piscinalis* | 0 | 0.6 ±4.7 | 0 | 0 | 0 | 0.2 ±2.4 |
| *Theodoxus fluviatilis* | 0 | 3.1 ±23.5 | 2.5 ±8.7 | 0 | 0 | 0.4 ±6.6 |
| *Potamopyrgus antipodarum* | 0 | 0 | 0 | 0 | 0 | 0.2 ±4.5 |
| *Hydrobia ulvae* | 0 | 0 | 0 | 0 | 0 | 0.4 ±6.6 |
| *Dreissena polymorpha* | 0 | 0 | 0.5 ±2.9 | 0 | 0.5 ±2.9 | 0.2 ±2.4 |
| *Pisidium* *amnicum* | 0 | 2.8 ±18.8 | 0 | 0 | 0 | 0 |
| *Unio tumidus* | 0 | 0 | 0 | 1.0 ±3.2 | 0 | 0 |
| *Unio pictorum* | 0 | 1.9 ±9.4 | 0 | 0 | 0 | 0 |
| *Anodonta anatina* | 0 | 0 | 0 | 1.5 ±6.2 | 0 | 0.4 ±5.9 |
