# Supplementary material for: Patterns of salinity regime in coastal lakes based on structure of benthic invertebrates
Source: PLoS One. 2018 Nov 26;13(11):e0207825. doi: 10.1371/journal.pone.0207825 (PMC6257944; doi:10.1371/journal.pone.0207825)
Supplement: S2 Table — (DOCX) [file pone.0207825.s002.docx]

**S2. Results of assessment based on the Indicator Value (IndVal) method for habitat types vs. all taxa, S.D. – standard deviation**

| **Name of taxa** | **Max. habitat** | **Observed IndVal** | **Indicator Value**  **from randomized groups** | | ***p*(MC)** |
| --- | --- | --- | --- | --- | --- |
|  |  |  | **Mean** | **S.D.** |  |
| OLIGOCHAETA | Brackish | 51.3 | 29.3 | 3.27 | **0.0002** |
| *Hediste diversicolor* | Brackish | 3.1 | 1.7 | 0.95 | 0.1218 |
| *Pygospio elegans* | Brackish | 2.1 | 1.4 | 0.83 | 0.3483 |
| *Mysis mixta* | Brackish | 4.2 | 1.9 | 0.99 | 0.0412 |
| *Asellus aquaticus* | Brackish | 4.2 | 2.0 | 1.06 | 0.0452 |
| *Gammarus debeni* | Brackish | 15.3 | 7.3 | 2.25 | **0.0054** |
| *Gammarus oceanicus* | Brackish | 4.8 | 2.7 | 1.31 | 0.0406 |
| *Corophium volutator* | Brackish | 7.3 | 3.0 | 1.41 | **0.0134** |
| *Idotea balthica* | Brackish | 1.0 | 1.3 | 0.25 | 1.0000 |
| *Neomysis integer* | Brackish | 9.4 | 3.1 | 1.23 | **0.0010** |
| *Glossiphonia complanata* | Transitional | 1.7 | 1.3 | 0.25 | 0.2547 |
| *Erpobdella octoculata* | Brackish | 0.7 | 1.5 | 0.78 | 1.0000 |
| *Pisicola geometra* | Brackish | 2.1 | 1.4 | 0.82 | 0.3363 |
| *Chironomus plumosus* | Brackish | 50.3 | 23.3 | 2.88 | **0.0002** |
| Chironomidae n.det. | Brackish | 12.2 | 6.8 | 1.81 | **0.0146** |
| *Dicrochironomus* sp. | Brackish | 6.1 | 3.2 | 1.33 | 0.0408 |
| *Procladius* sp. | Transitional | 14.4 | 11.9 | 2.62 | 0.1638 |
| *Polypedilum* sp. | Freshwater | 18.2 | 12.4 | 3.26 | 0.0418 |
| *Psectrocladius* sp. | Transitional | 3.4 | 4.8 | 1.82 | 0.7682 |
| *Bezzia* sp. | Brackish | 13.8 | 9.7 | 2.68 | 0.0422 |
| *Microtendipes* sp. | Brackish | 1.0 | 1.3 | 0.25 | 1.0000 |
| *Sergentia* sp. | Freshwater | 12.2 | 8.7 | 2.05 | 0.0422 |
| *Einfeldia* sp. | Brackish | 6.1 | 3.7 | 1.49 | 0.0464 |
| *Clunio* sp. | Brackish | 2.1 | 1.4 | 0.81 | 0.3369 |
| *Pelopia* sp. | Freshwater | 1.0 | 1.9 | 0.95 | 0.8282 |
| *Chaoborus* sp. | Freshwater | 1.3 | 1.3 | 0.25 | 0.5893 |
| *Diamesa campestris* | Transitional | 0.9 | 1.4 | 0.82 | 0.7249 |
| *Tabanus* sp. | Transitional | 1.7 | 1.3 | 0.25 | 0.2563 |
| *Corixa* sp. | Brackish | 2.1 | 1.4 | 0.83 | 0.3481 |
| *Ecnomus tenellus* | Freshwater | 1.3 | 1.3 | 0.25 | 0.5925 |
| *Limnephilus* sp. | Transitional | 1.0 | 1.7 | 0.93 | 0.8018 |
| *Caenis macrura* | Freshwater | 1.3 | 1.3 | 0.25 | 0.5775 |
| *Acentria ephemerella* | Brackish | 1.0 | 1.3 | 0.25 | 1.0000 |
| *Cataclysta lemnata* | Brackish | 1.0 | 1.3 | 0.25 | 1.0000 |
| *Bithynia tentaculata* | Brackish | 1.2 | 1.7 | 0.95 | 0.6691 |
| *Valvata piscinalis* | Freshwater | 1.8 | 1.7 | 0.95 | 0.4013 |
| *Theodoxus fluviatilis* | Transitional | 1.8 | 2.4 | 1.17 | 0.6231 |
| *Potamopyrgus antipodarum* | Brackish | 1.0 | 1.3 | 0.25 | 1.0000 |
| *Hydrobia ulvae* | Brackish | 1.0 | 1.3 | 0.25 | 1.0000 |
| *Dreissena polymorpha* | Brackish | 1.2 | 1.7 | 0.93 | 0.7942 |
| *Pisidium amnicum* | Freshwater | 1.3 | 1.3 | 0.25 | 0.5879 |
| *Unio tumidus* | Transitional | 4.3 | 1.4 | 0.82 | 0.0448 |
| *Unio pictorum* | Freshwater | 2.6 | 1.4 | 0.83 | 0.1848 |
| *Anodonta anatina* | Transitional | 2.4 | 2.0 | 1.01 | 0.2807 |
| Averages | | 8.8 | 6.6 | 0.90 | 0.3113 |
